# Enhanced C_30_ carotenoid production in *Bacillus subtilis* by systematic overexpression of MEP pathway genes

**DOI:** 10.1007/s00253-015-6531-3

**Published:** 2015-04-09

**Authors:** Dan Xue, Ingy I. Abdallah, Ilse E. M. de Haan, Mark J. J. B. Sibbald, Wim J. Quax

**Affiliations:** Department of Pharmaceutical Biology, GRIP, University of Groningen, Antonius Deusinglaan 1, 9713 AV Groningen, The Netherlands

**Keywords:** Terpenoids, MEP, *Bacillus subtilis*, Isoprene, Carotenoid

## Abstract

Creating novel biosynthetic pathways and modulating the synthesis of important compounds are one of the hallmarks of synthetic biology. Understanding the key parameters controlling the flux of chemicals throughout a metabolic pathway is one of the challenges ahead. Isoprenoids are the most functionally and structurally diverse group of natural products from which numerous medicines and relevant fine chemicals are derived. The well-characterized and broadly used production organism *Bacillus subtilis* forms an ideal background for creating and studying novel synthetic routes. In comparison to other bacteria, *B. subtilis* emits the volatile compound isoprene, the smallest representative of isoprenoids, in high concentrations and thus represents an interesting starting point for an isoprenoid cell factory. In this study, the effect of systematic overexpression of the genes involved in the methylerythritol phosphate (MEP) pathway on isoprenoid production in *B. subtilis* was investigated. *B. subtilis* strains harboring a plasmid containing C_30_ carotenoid synthetic genes, *crtM* and *crtN*, were combined with pHCMC04G plasmids carrying various synthetic operons of the MEP pathway genes. The levels of produced carotenoids, diaponeurosporene and diapolycopene, were used as indication of the role of the various enzymes on the flux of the MEP pathway. It was shown that the production of carotenoids can be increased significantly by overexpressing the MEP pathway enzymes. More broadly, the strains developed in this study can be used as a starting point for various isoprenoid cell factories.

**Electronic supplementary material:**

The online version of this article (doi:10.1007/s00253-015-6531-3) contains supplementary material, which is available to authorized users.

## Introduction

Isoprenoids, also known as terpenoids, represent the most functionally and structurally diverse group of natural products. They are found widespread among living organisms. Numerous isoprenoids have attracted commercial interest as pharmaceuticals or nutraceuticals. For example, isoprenoids extracted from plants are used as anticancer (paclitaxel) and antimalarial (artemisinin) drugs, whereas volatile monoterpenes are used as flavors and fragrances (Wallaart et al. 1999). Most isoprenoids are naturally produced in small quantities, and purification from biological material suffers from low yields, impurities, and consumption of large amounts of natural resources. Moreover, chemical synthesis of the majority of isoprenoids is problematic and expensive due to the complexity of their molecules. Therefore, the need for alternative methods of production for important isoprenoids has been a pressing issue in the last few decades, and to face this challenge, researchers extensively explored microbial production of isoprenoids (Martin et al. 2003).

**Fig. 1:**
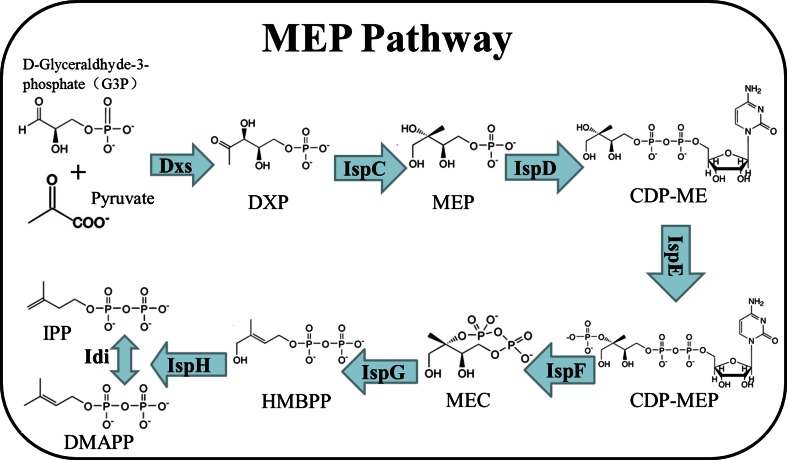
MEP pathway in *B. subtilis*. Eight enzymes are involved in the conversion of d-glyceraldehyde-3-phosphate and pyruvate to IPP and DMAPP. These two products are precursors for isoprenoid compounds such as carotenoids. Enzymes in the metabolic pathway: 1-deoxy-d-xylulose-5-phosphate synthase (*Dxs*), 1-deoxy-d-xylulose-5-phosphate reductoisomerase or 2-C-methyl-d-erythritol 4-phosphate synthase (Dxr, *IspC*), 2-C-methyl-d-erythritol 4-phosphate cytidylyltransferase (*IspD*), 4-(cytidine 5′-diphospho)-2-C-methyl-d-erythritol kinase (*IspE*), 2-C-methyl-d-erythritol 2,4-cyclodiphosphate synthase (*IspF*), (E)-4-hydroxy-3-methylbut-2-enyl-diphosphate synthase (*IspG*), 4-hydroxy-3-methylbut-2-enyl diphosphate reductase (*IspH*), and isopentenyl-diphosphate delta-isomerase (*Idi*). Intermediates in the metabolic pathway: 1-deoxy-d-xylulose 5-phosphate (*DXP*), 2-C-methyl-d-erythritol 4-phosphate (*MEP*), 4-(cytidine 5′-diphospho)-2-C-methyl-d-erythritol (*CDP-ME*), 2-phospho-4-(cytidine 5′-diphospho)-2-C-methyl-d-erythritol (*CDP-MEP*), 2-C-methyl-d-erythritol 2,4-cyclodiphosphate (*MEC*), (E)-4-hydroxy-3-methylbut-2-en-1-yl diphosphate (*HMBPP*), isopentenyl diphosphate (*IPP*), and dimethylallyl diphosphate (*DMAPP*)

Despite the great diversity of their structures, all isoprenoids are synthesized by consecutive condensation of two five-carbon precursors, isopentenyl diphosphate (IPP) and its isomer dimethylallyl diphosphate (DMAPP) (Julsing et al. 2006). For decades, the mevalonate (MVA) pathway was assumed to be the only source for IPP/DMAPP in all living organisms. However, it has been established nowadays that the MVA pathway is present in eukaryotes (the cytosol and mitochondria of plants), in archaea, and in some eubacteria (Bloch 1992; Smit and Mushegian 2000; Wilding et al. 2000). An alternative pathway for IPP/DMAPP formation, the 2-C-methyl-d-erythritol-4-phosphate (MEP) or 1-deoxy-d-xylulose-5-phosphate (DOXP) pathway (Fig. 1), was discovered recently to occur in eubacteria, algae, cyanobacteria, and the chloroplasts of plants (Eisenreich et al. 2004; Hunter 2007; Lichtenthaler 2000; Rohmer 1999).

There are many differences between the MVA and MEP pathways regarding stoichiometry, energy consumption, and the equilibrium between oxidation and reduction of glucose to IPP/DMAPP conversion (Steinbüchel 2003). The MVA pathway involves seven enzymes and starts by formation of acetoacetyl-CoA from two molecules of acetyl-CoA proceeding until the production of IPP/DMAPP. The MVA pathway has been extensively studied and introduced into heterologous hosts including *Escherichia coli* (Anthony et al. 2009; Martin et al. 2003; Rohdich et al. 2001). On the other hand, the MEP pathway utilizes pyruvate and glyceraldehyde-3-phosphate as starting materials. For *E. coli*, the complete MEP pathway has been elucidated, all genes involved have been determined, and their corresponding enzymes were described (Eisenreich et al. 2004). The story is different for *B. subtilis* where only a few studies have explored the MEP pathway leaving many questions yet to be answered (Julsing et al. 2007; Wagner et al. 2000; Xue and Ahring 2011).

*B. subtilis* contains an endogenous MEP pathway producing levels of isoprene higher than most other eubacteria including *E. coli* (Kuzma et al. 1995). *B. subtilis* has a broad metabolic potential, high growth rate, and wide substrate range. In addition, *B. subtilis* is listed by the Food and Drug Administration as generally regarded as safe (GRAS). These properties make *B. subtilis* an excellent candidate for the production of food grade or pharmaceutical isoprenoids by enhancing the flux of isoprenoid precursors via the MEP pathway. Some information regarding the improvement of the MEP pathway in *B. subtilis* has been reported (Julsing et al. 2007; Xue and Ahring 2011). The production of isoprenoids in *B. subtilis* has been limitedly studied where Yoshida et al. (2009) report the production of C_30_ carotenoids by expression of *CrtM* and *CrtN* genes. Also, overexpression of *dxs* and *idi* was shown to increase production of the C_15_ terpenoid amorphadiene in *B. subtilis* (Zhou et al. 2013). We chose to explore the engineering of endogenous MEP pathway in *B. subtilis* rather than the heterologous MVA pathway, as using endogenous genes requires less genetic adaptations and has a higher chance of producing correctly folded proteins and, hence, success. In the current study, we aim at examining the effect of MEP pathway modulation on the production of isoprenoids in *B. subtilis*. Until now, only the effect of overexpression of *dxs*, *ispC*, and *idi* genes (in *B. subtilis* MEP pathway) on the overall production of isoprenoids has been reported, while the other enzymes in the pathway have yet to be explored. We describe the systematic analysis of a series of synthetic operons expressing most of the respective enzymes from the *B. subtilis* MEP pathway. The level of production of C_30_ carotenoids was used as a read out to assess the effect of such modulations. This study can provide a basis for further fine tuning of the MEP pathway in *B. subtilis* aiming at a more efficient production of isoprenoids.

## Materials and methods

### Bacterial strains, plasmids, and growth conditions

Bacterial strains and plasmids used in this study are listed in Table 1. Experiments with *E. coli* DH5α strains were performed using Luria-Bertani broth (LB) while those with *B. subtilis* 168 strains were performed using tryptic soy broth (TSB) (17 g/l tryptone, 3 g/l soytone, 2.5 g/l dextrose, 5.0 g/l NaCl, 2.5 g/l K_2_HPO_4_). Both *E. coli* and *B. subtilis* 168 were grown at 37 °C under shaking conditions. When necessary, growth media were supplemented with antibiotics in the following concentrations: 10 μg/ml chloramphenicol, 100 μg/ml ampicillin, or 100 μg/ml erythromycin for *E. coli* DH5α and 5 μg/ml chloramphenicol or 20 μg/ml tetracycline for *B. subtilis* 168.

**Table 1:** Bacterial strains and plasmids used in this study

Bacterial strain	Genotype	Reference
*B. subtilis* 168	*trpC2*	Kunst et al. (1997); Barbe et al. (2009)
*E. coli* DH5α	F^−^ *end*A1 *hsd*R17 (r_k_^−^,m_k_^+^) *sup*E44 *thi* ^−^1 λ^−^ *rec*A1 *gyr*A96 *rel*A1 φ80d*lac*Z∆M15	Bethesda Research Laboratories 1986
Plasmid	Relevant properties	Reference
pHB201	*B. subtilis* and *E. coli* shuttle vector; ori-pUC19; ori-pTA1060; P59 promoter; *cat86::lacZα*; Cm^R^; Em^R^	Bron et al. (1998)
pHCMC04G	*B. subtilis* and *E. coli* shuttle vector; ori-pBR322; ori-pBS72; P_*xylA*_xylose-inducible promoter; Cm^R^; Amp^R^	This study
pHYCrtMN	*B. subtilis* and *E. coli* shuttle vector; ori-pACYC177; ori-pAMα1; *crtM* and *crtN* genes of *S. aureus*; Amp^R^; Tc^R^	Yoshida et al. (2009)

### Construction of MEP pathway synthetic operons

The synthetic operons were constructed partly based on the procedure as described by Akhtar and Jones (2008). Primers to amplify the genes are listed in Table S1.

Eight synthetic operons, each containing from one to four genes of the MEP pathway, were constructed. The first gene (*dxs*) was amplified using a forward primer that contains a *Spe*I restriction site and the *B. subtilis mntA* ribosomal binding site (RBS) plus linker (AAGAGGAGGAGAAAT) and a reverse primer that contains a linker with *Swa*I and *Bgl*II restriction sites, a 6× His-tag, a stop codon, and a *Bam*HI restriction site (Fig. S1). The amplified product was cloned into the pHB201 plasmid via *Spe*I and *Bam*HI sites. The consecutive genes were cloned as follows: The next gene to be introduced was amplified with a forward primer containing a *Stu*I restriction site, a stop codon for the previous gene, and the *B. subtilis mntA* RBS plus linker, and a reverse primer containing a linker with *Swa*I and *Bgl*II restriction sites. The amplified gene was then cut at the *Stu*I and *Bgl*II restriction sites and cloned into the pHB201-dxs constructed plasmid cut with *Swa*I and *Bgl*II restriction enzymes. This procedure was repeated until up to four genes were cloned into the pHB201 plasmid. Based on the gene sequences, a silent mutation was performed whenever necessary or a restriction site for *Sma*I was used instead of *Stu*I.

The synthetic operons were cut from the pHB201 plasmid with restriction enzymes *Spe*I and *Bam*HI (see Fig. S1) and introduced into the *Spe*I/*Bam*HI-restricted pHCMC04G plasmid constructed in this study (see supplementary material and Fig. S2). The pHCMC04G plasmid has a θ-replication system, making it more stable than the pHB201 plasmid; hence, it will be used for expression in *B. subtilis* 168. The resulting constructs are listed in Table 2, and the sequences of all the plasmids were confirmed by sequencing (Macrogen, Europe). The pHCMC04G constructs were chosen to transform competent *B. subtilis* 168 cells as described by Kunst and Rapoport (1995).

**Table 2:** Plasmid constructs used for expression in *B. subtilis*

Plasmid	Vector	MEP pathway genes
p04S	pHCMC04G	*dxs*
p04SD	pHCMC04G	*dxs + ispD*
p04SDF	pHCMC04G	*dxs + ispD + ispF*
p04SDFH	pHCMC04G	*dxs + ispD + ispF + ispH*
p04C	pHCMC04G	*ispC*
p04CE	pHCMC04G	*ispC + ispE*
p04CEG	pHCMC04G	*ispC + ispE + ispG*
p04CEGA	pHCMC04G	*ispC + ispE + ispG + ispA*

### Expression of MEP pathway genes

To check for the production of MEP pathway enzymes, *B. subtilis* 168 strains with pHCMC04G constructs were grown in 50 ml TSB medium containing appropriate antibiotics. Overnight cultures were diluted to an optical density at 600 nm (OD_600_) of 0.05 in TSB medium. The cultures were grown for 3 h at 37 °C under shaking conditions (250 rpm). Then, xylose was added to a final concentration of 1 % to start induction. The cultures were grown overnight before protein expression analysis was done. Cells were collected by centrifugation, 30 min at 2100 *g*, 4 °C, and resuspended in 2 ml lysis buffer (25 mM Tris-HCl, pH 8.0, 25 mM NaCl, 20 % glucose, and 0.25 mg/ml lysozyme). Cells were lysed by incubation at 37 °C for 30 min, and the soluble protein fractions were collected by centrifugation, 20 min at 17,000 *g*. Proteins were then purified using HisSpinTrap™ columns (GE Healthcare) using 25 mM Tris-HCl, 500 mM NaCl, and 20 mM imidazole, pH 7.4, as equilibration and wash buffer and 25 mM Tris-HCl, 500 mM NaCl, and 500 mM imidazole, pH 7.4, as elution buffer and were analyzed by SDS-PAGE using precast NuPAGE® gels (Invitrogen) and Western blotting. For Western blotting, the presence of His-tagged proteins was monitored by immunodetection with specific antibodies against the His-tag. Primary bound antibodies were visualized using fluorescent IgG secondary antibodies (IRDye800 CW goat anti-rabbit LiCor Biosciences). Membranes were scanned for fluorescence at 800 nm using the Odyssey Infrared Imaging System (LiCor Biosciences).

### Introduction of pHYcrtMN in *B. subtilis* 168 cells overexpressing MEP pathway genes

*B. subtilis* 168 cells containing the pHCMC04G plasmids with the synthetic operons were transformed with the pHYcrtMN plasmid (Yoshida et al. 2009) as described before by Kunst and Rapoport (1995) and plated on LB agar plates containing chloramphenicol and tetracycline. Yellow colonies were picked for extraction and analysis of the produced carotenoids.

### Extraction of carotenoids from *B. subtilis* 168

Carotenoids were extracted from *B. subtilis* 168 cells as described before by Yoshida et al. (2009) with a few modifications. Overnight cultures of *B. subtilis* 168 strains containing pHYcrtMN and pHCMC04G with synthetic MEP operons were inoculated in 50 ml TSB at OD_600_ 0.05. Then, 1 % xylose was added for induction after 3 h and the strains were cultured for a total of 24 h at 37 °C (250 rpm). Samples were then centrifuged at 2100 *g* for 30 min, and pellets were washed with 1 ml TE (10 mM Tris, 1 mM EDTA). The cells were resuspended in 200 μl TE, and 20 μl squalene (0.97 mg/ml, dissolved in isopropanol) was added as an internal standard. To extract the carotenoids, cells were lysed by adding 50 μl of 20 mg/ml lysozyme and incubating for 15 min at 37 °C. Cell lysates were then transferred into glass tubes, covered in aluminum foil to avoid light exposure, and centrifuged for 20 min at 2100 *g*. After discarding the supernatant, 1 ml acetone was added to the pellets and they were vortexed for 2 min. The samples were incubated at 55 °C for 1 min then vortexed for 1 min, followed by centrifugation for 10 min at 2100 *g*. The supernatants were transferred to new glass tubes. The acetone extraction was repeated four times. Then, the acetone extracts were evaporated and the remaining carotenoids were dissolved in 100 μl acetone and collected in HPLC vials. The extracts were used for HPLC analysis. All strains were cultured in triplicates under the same conditions, and the extraction of each sample was performed in parallel. Cell dry weight was determined by pelleting and drying a fraction of the culture.

### Carotenoid isolation and identification

Isolation of the carotenoids was carried out by preparative HPLC (Millipore) using a C_18_ column (Phenomenex Luna 5 μm, 100 Å, 250 × 21.20 mm). Acetone extracts were loaded on the column and eluted by acetonitrile and H_2_O (92:8 %) at a flow rate of 8 ml/min. The carotenoids were eluted at 38 and 41 min and then identified by UV spectrum and liquid chromatography/mass spectrometry (LC/MS) analysis. Purified carotenoids were used as standard compounds for quantitative analysis.

### HPLC analysis of carotenoids from *B. subtilis* 168

All samples were analyzed with Shimadzu HPLC system equipped with a Kinetex C_18_ column (2.6 μm, 100 Å, 100 × 4.60 mm) at 25 °C with a diode array detector. Carotenoids were eluted with acetonitrile/water at 1 ml/min. Mobile phase A consists of 5 % acetonitrile, 95 % H_2_O, and 0.1 % trifluoroacetic acid (TFA). Mobile phase B consists of 100 % acetonitrile and 0.1 % TFA. The HPLC program was as follows: 0–15 min, 92 % mobile phase B; 16–30 min, 100 % mobile phase B; and 31–35 min, 92 % mobile phase B to equilibrate the column for next run. Injection volume was 20 μl, and detection was carried out at 450 nm for carotenoids and 200 nm for squalene. The results were calibrated by the internal standard squalene, and the carotenoids produced by all strains were quantified using carotenoid standard curves. The *B. subtilis* strain that only contains the pHYcrtMN plasmid was used as a control strain.

### Nucleotide sequence accession number

The nucleotide sequence of the complete genome of *Bacillus subtilis* 168 was awarded the following accession numbers: AL009126 and NC000964. All genes used in this study were amplified from the genomic DNA of this strain.

## Results

### A synthetic operon for coordinated expression of MEP pathway genes can be stably maintained in *B. subtilis* 168

**Fig. 2:**
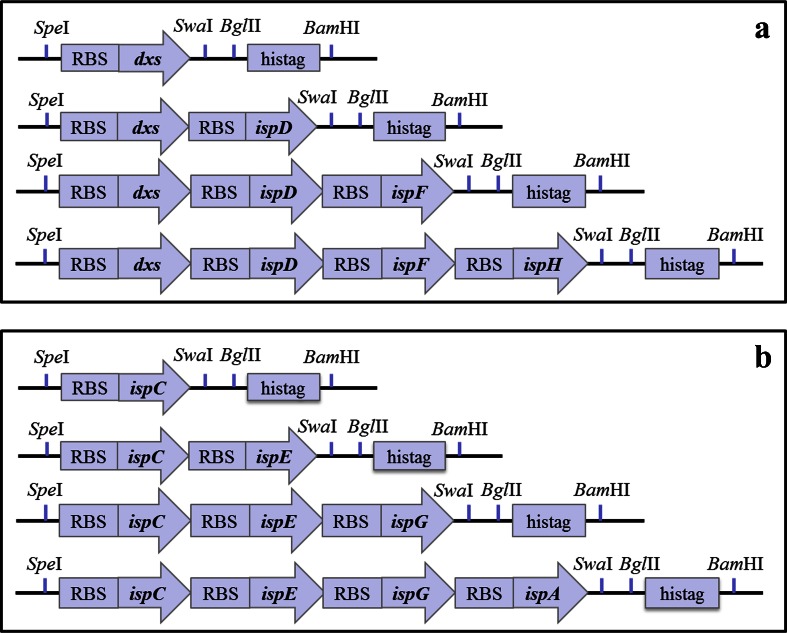
Constructed operons. **a** Operons constructed with MEP pathway genes of group A. **b** Operons constructed with MEP pathway genes of group B

To investigate the possible increase of the intracellular concentration of isoprenoids, we assembled the endogenous genes of *B. subtilis* 168 encoding the MEP pathway in a synthetic operon that is expressed from a plasmid. We divided the genes in two groups, referred to as “A” and “B” from here on (Fig. 2). The division is based on the results obtained from previous research in our lab (Julsing et al. 2007), where the effect of the MEP pathway genes on isoprene production was studied using *B. subtilis* 168 single conditional mutants. In that study, the *dxs*, *ispD*, *ispF*, and *ispH* mutants showed, at minimal required induction for survival, the largest decrease in isoprene production and might therefore represent the catalysts with the largest control on the flux in the pathway leading to isoprenoid production. Similarly, Yuan and colleagues found in *E. coli* that when *dxs*, *ispD*, and *ispF* were overexpressed by using a strong bacteriophage promoter, the production of *β*-carotene was increased in these strains (Yuan et al. 2006). Thus, the *dxs*, *ispD*, *ispF*, and *ispH* genes represent group A. Group B contains the other three genes of the MEP pathway—*ispC*, *ispE*, and *ispG—*and the downstream gene *ispA*(*yqiD*) that codes for a farnesyl diphosphate synthase. The latter enzyme catalyzes the formation of geranyl diphosphate (GPP) and farnesyl diphosphate (FPP) leading to geranylgeranyl diphosphate (GGPP) from the precursors IPP/DMAPP. The *idi* gene (the final gene in the MEP pathway encoding for an IPP isomerase) was not chosen in construction of the operons based on indications that it is non-essential in *B. subtilis* (Takagi et al. 2004). Previous research also showed that a knockout *B. subtilis* strain of the *ypgA* gene encoding *idi* is viable and produces isoprene. The isoprene production implies that the synthesis of the isomer DMAPP does not rely on the isomerase (Eisenreich et al. 2004; Julsing et al. 2007). Synthetic operons were constructed by first cloning into pHB201 then transferring to pHCMC04G resulting in eight different pHCMC04G constructs. The constructs were initially assembled in *E. coli* and subsequently transferred to *B. subtilis* 168 where they were found to be stably maintained.

### All MEP pathway enzymes can be overexpressed in *B. subtilis* 168

In order to monitor expression of the consecutive MEP pathway genes, cell lysates were enriched for His-tagged proteins and analyzed by Western blotting. In our design, only the last protein carries a His-tag. As all genes on the plasmid are in one operon and were checked by sequencing, it can be concluded that the transcripts are intact whenever this last protein is visible. This strategy was preferred over one where all proteins contain a His-tag, as addition of any sequence, and thus, also a His-tag may have an influence on the folding and/or activity of a protein. All enzymes from the MEP pathway can be detected on the Western blot proving that they are overexpressed in *B. subtilis* 168.

### Carotenoid production is highly increased in MEP pathway-engineered *B. subtilis* 168 strains

**Fig. 3:**
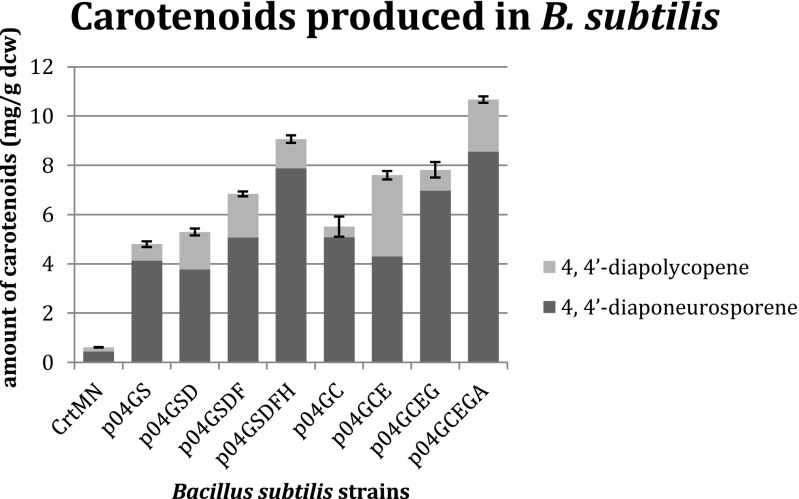
Quantitative analyses of carotenoids produced in *B. subtilis* 168 strains overexpressing MEP pathway enzymes. Carotenoids were extracted from *B. subtilis* 168 cells with acetone. Samples were analyzed by HPLC. Individual and total amount of carotenoids were calibrated by the internal standard squalene and determined using carotenoid standard curves. Shown are the amounts of produced carotenoids in strains overexpressing MEP pathway enzymes from the pHCMC04G plasmid per gram dry cell weight (±standard deviation). The amount of diaponeurosporene is indicated in *black* and the amount of diapolycopene in *gray*. The experiments were performed in triplicate

A comparison of carotenoid production in the different strains of modified *B. subtilis* was performed where carotenoids were quantified by HPLC. The *B. subtilis* strain that only contains the pHYcrtMN plasmid was used as a control strain. The chromatograms show two major peaks at 450 nm (Fig. S3). The first peak, with maximal absorbance at 443, 472, and 501 nm, eluted at 8.5 min. The mass spectra of this peak showed [M + H]^+^ at *m*/*z* 401, which is in agreement with the molecular formula C_30_H_40_ of 4,4′-diapolycopene (Figs. S3 and S4a). The second peak eluting at 10.5 min was identical to that of 4,4′-diaponeurosporene as its absorption maxima were at 415, 439, and 469 nm, and the mass spectra showed [M + H]^+^ at *m*/*z* 403 which is in accord with the formula C_30_H_42_ (Figs. S3 and S4b) (Takaichi 2000; Takaichi et al. 1997). Squalene was detected at 200 nm and eluted from the column at 24.5 min. The biosynthetic pathway involves conversion of 4,4′-diaponeurosporene to 4,4′-diapolycopene in a reaction catalyzed by *CrtN* (Wieland et al. 1994). Literature shows that *CrtN* has far less activity than *CrtM* in *Staphylococcus aureus*. Wild-type *S. aureus* does not produce any 4,4′-diapolycopene, and in mutants where the downstream pathway from 4,4′-diaponeurosporene to staphyloxanthin was blocked, only a small amount of 4,4′-diapolycopene was produced (Marshall and Wilmoth 1981a; Marshall and Wilmoth 1981b). It is apparent that this is also the case in *B. subtilis* 168 since peaks for both 4,4′-diaponeurosporene and 4,4′-diapolycopene were present in the chromatograms of all samples. Thus, the two compounds were calculated individually and together as total carotenoids. The results are shown in Fig. 3 and Table 3. The sum of the two carotenoids was quite constant between replicates as evidenced by the low standard deviations.

**Table 3:** Amount of carotenoids produced by engineered *B. subtilis* strains and relative increase compared to control strain

*B. subtilis* strains	Total carotenoids (mg/g dcw)^a^	Relative increase^b^
CrtMN	0.60(±0.010)	1.00
p04S	4.81(±0.114)	8.02
p04SD	5.27(±0.140)	8.78
p04SDF	6.83(±0.096)	11.38
p04SDFH	9.03(±0.158)	15.05
p04C	5.54(±0.410)	9.23
p04CE	7.62(±0.176)	12.70
p04CEG	7.84(±0.315)	13.07
p04GCEGA	10.65(±0.129)	17.75

^a^The total amount of carotenoids was measured in triplicate (±standard deviation)

^b^The relative increase is calculated as the amount of carotenoids produced in the engineered *B. subtilis* strain divided by the amount of carotenoids produced in the *B. subtilis* strain containing only the carotenoids producing plasmid pHYCrtMN

*B. subtilis* strain p04S which carries *dxs* shows a total carotenoid level of 4.81 (±0.114) mg/g dry cell weight (dcw), which is an 8-fold increase in total carotenoid production compared to the control strain (Fig. 3 and Table 3). In strain p04SD overexpressing *dxs* and *ispD*, production of carotenoids was increased by 8.7-fold compared to the control strain. The level of carotenoid production is slightly increased compared to strain p04S, suggesting that *ispD* has a low contribution to the overall flux of the MEP pathway, thus not considered a bottleneck in production of isoprenoids. Additional overexpression of *ispF* together with *dxs* and *ispD* in strain p04SDF resulted in an 11.4 times elevation in carotenoid production compared to the control strain. This implies that *ispF* is important for an increase in the pathway flux. Finally, in strain p04SDFH expressing all group A genes, the yield of carotenoids was increased by 15-fold relative to the control strain.

Regarding the strains with group B operon, carotenoid production from strain p04C harboring *ispC*(*dxr*) was 9.2-fold higher compared to the control strain. This shows for the first time the importance of overexpressing *ispC*, which was not reported in previous studies. The level of isoprene production was previously reported to be not significantly changed in the *B. subtilis* strain overproducing *ispC* (Xue and Ahring 2011). In strains p04CE and p04CEG, carotenoid production concurrently showed a 12.7-fold and 13.1-fold increase compared to the control strain. The highest total amount of carotenoids was obtained with the strain that overexpressed all group B genes: *ispC*, *ispE*, *ispG*, and *ispA* (strain p04CEGA). Additional overexpression of *ispA* in strain p04CEGA improved the production of total carotenoids by 17.8-fold (10.65 mg/g dcw) compared to the control strain.

## Discussion

Research in recent years has been directed toward elucidating the pathways leading to IPP and DMAPP, the building blocks in the biosynthesis of numerous natural products. Most studies have focused on the MVA pathway and on improving the flux through it both in natural hosts as in heterologous hosts. Recently, the attention has been shifted toward the MEP pathway endogenous to many eubacteria.

In this work, we have focused on the systematic analysis of the consecutive enzymes constituting the MEP pathway in *B. subtilis*. In a follow-up to our previous systematic deletion study of genes encoding MEP pathway enzymes (Julsing et al. 2007), we now have constructed synthetic operons encoding combinations of genes for overexpression of MEP pathway enzymes. As it had already been shown that the endogenous MEP pathway can be used for carotenoid production in *B. subtilis* 168 (Yoshida et al. 2009), we decided to use a C_30_ carotenoid-producing plasmid as a quantitative readout system for measuring the flux to isoprenoid production. From our studies, it is clear that an increase in the formation of carotenoids occurs when the level of isoprenoid precursors in *B. subtilis* 168 cells is elevated by engineering the MEP pathway. In order to unravel the individual contribution of each of the enzymes, *B. subtilis* 168 strains where transformed with synthetic operons harboring stepwise augmented combinations of the MEP pathway enzymes encoding genes on a plasmid. The vector used for gene expression was the pHCMC04G plasmid with a θ-replication system, in which expression is controlled by the xylose-inducible P_xylA_ promoter. As can be seen from Fig. 3, each insertion of an additional gene from group A or from group B leads to a higher production of carotenoids. This implies that almost each of the enzymes has, to varying extents, a control on the flux through the MEP pathway.

Overexpression of *dxs* showed a high increase in carotenoid yield (8-fold). This is in agreement with previous findings that overexpression of *dxs* results in higher amounts of isoprenoids in *B. subtilis* 168 (Xue and Ahring 2011; Zhou et al. 2013), although the extent of the increase in our study is much higher. Also, the strain overexpressing *ispC*(*dxr*) from plasmid p04C shows a 9.2-fold increase in carotenoid production. Previous studies in *B. subtilis* suggested that the amount of DXR is not a limiting step for isoprenoid biosynthesis (Xue and Ahring 2011). However, it has been reported in *E. coli* that the effect of *ispC* overexpression on MEP pathway flux can vary (Kim and Keasling 2001).

Each consecutive expression of an additional enzyme involved in the MEP pathway resulted in a higher amount of carotenoids detected. In group A synthetic operons, the limited increase in strain p04SD indicates that *ispD* is not a rate limiting step in the MEP pathway, at least not at the current production levels. For the same reason, as additional expression of *ispF* increased the level of produced carotenoids significantly, it is suggested that *ispF* has a larger contribution to the flux control in the MEP pathway than *ispD*. Additional insertion of gene *ispH* (p04SDFH) yields 9.03 (±0.16) mg/g dcw carotenoids, which is 30 % higher compared to strain p04SDF and a 15-fold increase over the control strain, demonstrating that the flux of isoprenoids can be further improved by enhancing *ispH* expression. The highest amount of produced carotenoids was observed in the strain overexpressing all group B enzymes (p04CEGA), which produces 10.65 (±0.13) mg/g dcw carotenoids representing ~18-fold increase over wild-type *B. subtilis*. This large increase is mainly caused by the major influence of *ispC* on the carotenoid production that was not anticipated based on previous reports. It is possible that the *B. subtilis* 168 strain used by Xue and Ahring (2011) exhibits a higher endogenous *ispC* expression level than the one used by us; hence, their overexpression of *ispC* has limited impact on isoprene production. Within the framework of the *Bacillus* functional analysis project, it has been noted that even after limited passages, the *B. subtilis* 168-type strain does acquire multiple mutations that can alter the phenotype considerably (Barbe et al. 2009).

Our findings concerning the level of increase in carotenoid production were compared with the level of decrease in isoprene production observed in the previous knockout study in *B. subtilis* (Julsing et al. 2007). Interestingly, strains overexpressing *ispC*, *ispE*, *ispF*, and *ispH* showed the expected effect on formation of the final products, to an extent that is in accord with the corresponding knockout strains. *Dxs* has a significant influence on the end products of both *dxs* knockout strain and overexpression strain (p04S), so this further confirms the key role of *dxs* in the MEP pathway. In the *ispD* knockout strain, a large decrease in isoprene production was observed. However, the amount of carotenoids produced in the strain additionally overexpressing *ispD* (p04SD) was only 1.1-fold higher than that in strain p04S. This may be indicative of the fact that *ispD* is essential for isoprenoid production, but its contribution to the flux control is low. Also, the increase in carotenoid production with overexpression of the rest of the MEP pathway enzymes is in concert with the effect of the corresponding knockout strains.

In conclusion, the observed enhancement of carotenoid production in the engineered *B. subtilis* 168 indicates that MEP pathway modulation shows a great promise for the production of isoprenoids. The engineered *B. subtilis* 168 strains provided by this study in combination with other terpene synthases represent an opportunity for a GRAS cell factory for the production of numerous isoprenoids.

## Electronic supplementary material

Below is the link to the electronic supplementary material.

ESM 1 (PDF 360 kb)
