# Supplementary material for: Enhanced C30 carotenoid production in Bacillus subtilis by systematic overexpression of MEP pathway genes
Source: Appl Microbiol Biotechnol. 2015 Apr 9;99(14):5907–15. doi: 10.1007/s00253-015-6531-3 (PMC4480331; doi:10.1007/s00253-015-6531-3)
Supplement: Supplementary file 1 — (PDF 360 kb) [file 253_2015_6531_MOESM1_ESM.pdf]

Supplementary material

Applied Microbiology and Biotechnology

Enhanced C_{30} carotenoids production in *Bacillus subtilis* by systematic overexpression of MEP pathway genes

Dan Xue[1], Ingy I. Abdallah[1], Ilse E.M. de Haan[1], Mark J.J.B. Sibbald[1], Wim J. Quax[1*]

[1] Department of Pharmaceutical Biology, GRIP, University of Groningen, Antonius Deusinglaan 1, 9713 AV, Groningen, The Netherlands

* Corresponding author: Department of Pharmaceutical Biology, University of Groningen, Antonius Deusinglaan 1, 9713 AV, Groningen, The Netherlands Tel. +31-50-3632558, Fax. +31-50-3633000, E-mail: w.j.quax@rug.nl

Keywords: terpenoids, MEP, *Bacillus subtilis*, isoprene, carotenoid

Materials and methods

Construction of plasmid pHCMC04G

The pHB201 plasmid replicates via the rolling circle replication system (Gruss and Ehrlich 1989). It is known that vectors based on this replication system can be unstable when large fragments of DNA are inserted into these plasmids (Bron et al 1985, Bron et al 1988). To overcome the problem of instability, plasmids with a θ-replication system can be used. This is a more stable system than the rolling circle replication system. This θ-replication system is found in larger *B. subtilis* plasmids (>50 kb) such as pLS20 (Meijer et al. 1995) and pBS72 (Titok et al. 2003). Based on pBS72, several θ-replicating *E. coli* / *B. subtilis* shuttle vectors have been constructed (Titok et al 2003, Nguyen et al 2005). One of them is the low copy number pHCMC04 plasmid, in which expression is controlled by the xylose-inducible P_{xylA} promoter.

All genes that are added in the synthetic operon contain the *B. subtilis mntA* RBS. However upstream of the *Spe*I restriction site, which is used to introduce the operon in the vector, the end of the P_{xylA} promoter sequence in pHCMC04 serves as an additional RBS, followed by a start codon without a stop codon (Fig. S2). To avoid an additional translation of the downstream gene, we constructed plasmid pHCMC04G. A stop codon was introduced between the start codon and the *Spe*I restriction site in plasmid pHCMC04 (obtained from Bacillus Genetic Stock Center, ECE189) by using MEGAWHOP PCR (Miyazaki 2011). Primers pHCMC04G_F and pHCMC04_R for constructing the megaprimer are listed in Table S1. Plasmid pHCMC04G was confirmed by sequencing.

References

Bron S, Luxen E (1985) Segregational instability of pUB110-derived recombinant plasmids in *Bacillus subtilis*. Plasmid 14(3):235-44

Bron S, Luxen E, Swart P (1988) Instability of recombinant pUB110 plasmids in *Bacillus subtilis*: plasmid-encodes stability function and effects of DNA inserts. Plasmid 19(3):231-41

Gruss A, Ehrlich SD (1989) The family of highly interrelated single-stranded deoxyribonucleic acid plasmids. Microbiol Rev 53(2):231-41

Meijer WJ, de Boer AJ, Van Tongeren S, Venema G, Bron S (1995) Characterization of the replication region of the *Bacillus subtilis* plasmid pLS20: a novel type of replicon. Nucleic Acids Res. 23(16):3214–23

Miyazaki K (2011) Chapter seventeen - MEGAWHOP Cloning: A Method of Creating Random Mutagenesis Libraries via Megaprimer PCR of Whole Plasmids. In: Christopher V (ed) Methods in Enzymology. vol Volume 498. Academic Press, pp 399-406

Nguyen HD, Nguyen QA, Ferreira RC, Ferreira LC, Tran LT, Schumann W (2005) Construction of plasmid-based expression vectors for *Bacillus subtilis* exhibiting full structural stability. Plasmid 54(3):241-248

Titok MA, Chapuis J, Selezneva YV, Lagodich AV, Prokulevich VA, Ehrlich SD, Jannière L (2003) *Bacillus subtilis* soil isolates: plasmid replicon analysis and construction of a new theta-replicating vector. Plasmid 49(1):53-62

Figure Legends

Fig. S1. Strategy for constructing the synthetic operons. The first gene, *dxs*, was cloned in the pHB201 plasmid using *Spe*I and *Bam*HI restriction sites. The inserted gene contains the *B. subtilis mntA* RBS, a linker containing *Swa*I and *Bgl*II restriction sites, a His-tag and a stop codon. The next gene, *ispD*, was cloned in the *Swa*I and *Bgl*II restriction sites, thereby destroying the *Swa*I restriction site. This was continued until all genes were in the synthetic operon. The last gene that is introduced contains the His-tag and expression can be checked by western blotting with antibodies against the His-tag.

Fig. S2. Schematic representation of the region of plasmid pHCMC04 that was modified to obtain plasmid pHCMC04G. P_{xylA} is the xylose-inducible promoter; the additional RBS is highlighted; the mutation is depicted in **bold**; the *SpeI* restriction site in *italics*; and the start codon that cause the problem is underlined.

Fig. S3. Chromatogram of carotenoids separated by HPLC. The *B. subtilis* 168 strains with pHCMC04G plasmids containing genes from the MEP pathway were transformed with the carotenoids-producing plasmid pHYCrtMN and the relative amount of carotenoid compounds was determined using HPLC with squalene as an internal standard.The two major peaks correspond to 4,4'-diapolycopene (1) and 4,4'-diaponeurosporene (2) (black line, 450 nm). Squalene was used as an internal standard (3) (grey line, 200 nm). (**a**) Chromatogram of the control strain (*B. subtilis* 168 with the pHYcrtMN plasmid). (**b**) Chromatogram of *B. subtilis* 168 pHYcrtMN overexpressing *dxs*. (**c**) UV-Visible spectrum of carotenoid extract peak 1, 4,4'-diapolycopene. (**d**) UV-Visible spectrum of carotenoid extract peak 2, 4,4'-diaponeurosporene.

Fig. S4. LC/MS analysis of carotenoids extracts. (**a**) Spectrum of peak 1 with pos APPI using F-benzene as dopant Mol weight 400: $M^{+\bullet}$: m/z 400.2 and$[M+H]^+$: m/z 401.2, identified as 4,4'-diapolycopene. (**b**) Spectrum of peak 2 with pos APPI using F-benzene as dopant, Mol weight 402: $M^{+\bullet}$: m/z 402.2 and$[M+H]^+$: m/z 403.2, identified as 4,4'-diaponeurosporene.

Table S1. Primers used to amplify the MEP pathway genes

Primer name	Sequence(5’→3’)
dxs-F3	GGGAGGCACTAGTAAGAGGAGGAGAAATATGGATCTTTTATC
dxs-R3	CGCGGATCC**TCA***GTGATGATGATGATGATG*CAGATCTGCATTTAAATGTGATCCAATTCCTTTGTG
ispD-F3	GGGACCGAAGGCCTG**TAA**GCCAAGAGGAGGAGAAATATGAGTTATGATGTGG
ispD-R3	CCCAGGCGCAGATCTCGATTTAAATGAACATGTTTATTCCCAC
ispF-F3	CGCACCCGGGC**TAA**CGCAAGAGGAGGAGAAATATGTTTAGAATTGGAC
ispF-R3	CCCAAGCGCAGATCTCGATTTAAATCGCCTTTTTGTATCAGTACTG
ispH-F3	GCGAAGGCCTC**TAA**CGCAAGAGGAGGAGAAATATGGACGTAATTAA
ispH-R3	GGCAGCGCCGCAGATCTCGATTTAAATGGTTTTTTGCTTTTACTTTTGG
ispC-F3	GGGAGGGGCACTAGTAAGAGGAGGAGAAATATGAAAAATATTTGTC
ispC-R3	CGCGGATCC**TCA***GTGATGATGATGATGATG*CAGATCTCGATTTAAATGTGTGAGTATTGAATT
ispE-F3	CCGAAGGCCTC**TAA**CGCAAGAGGAGGAGAAATATGCGTATTTTAG
ispE-R3	CGCAGATCTCGATTTAAATGATCAAGAGCGTTCTGTTCGCCG
ispG-F3	GCGCCCGGGC**TAA**CGCAAGAGGAGGAGAAATATGCAAGTGAGTG
ispG-R3	CGCAGATCTCGATTTAAATCAGCTTTTTGTGTTTCTTC
ispA-F3	GCGGAGGCCTC**TAA**CGCAAGAGGAGGAGAAATATGACAAATAAAT
ispA-R3	GCGCGCAGATCTCGATTTAAATCGTGATCTCTTGCCGC
pHCMC04G_F	CAAAGGGGGAAATGACAAATGGTC**TAA**ACTAGTGATATCTAAAAATCAAAGGG
pHCMC04G_R	TCTTTCCGAGCTTCGTCCAA

Restriction sites are underlined; His-tag sequence is indicated in *italics*; stop codons are indicated in **bold**; in each forward primer there is the ribosomal binding sequence of *B. subtilis mntA* (AAGAGGAGGAGAAAT).

Figure S1

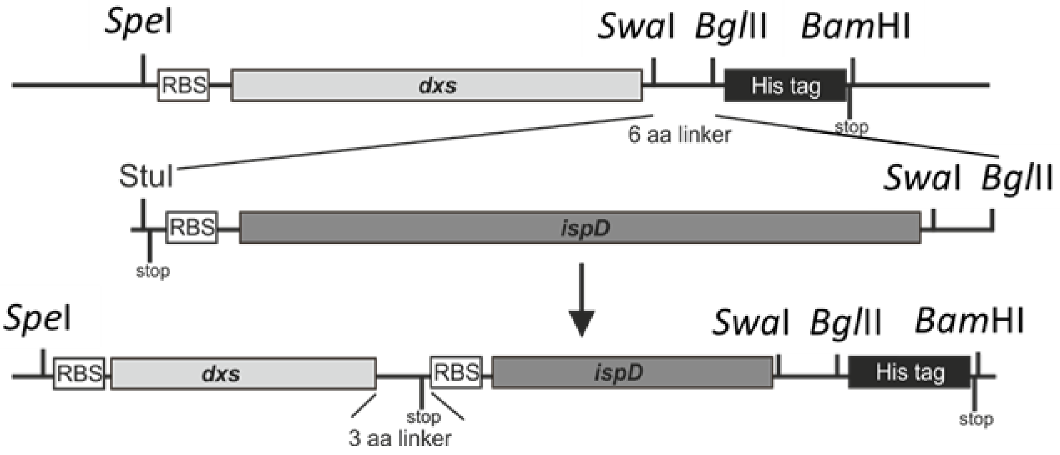
SpeI
SwaI
BglII
BamHI
RBS
dxs
His tag
stop
6 aa linker
StuI
SwaI
BglII
RBS
ispD
stop
SpeI
SwaI
BglII
BamHI
RBS
dxs
RBS
ispD
His tag
stop
3 aa linker
stop

Figure S2

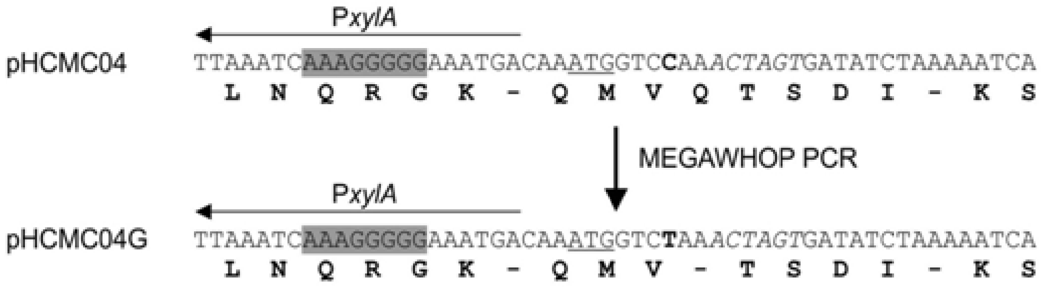

PxylA
pHCMC04
TTAAATCAAAGGGGGAAATGACAAATGGTCCAAACTAGTGATATCTAAAAATCA
L N Q R G K - Q M V Q T S D I - K S
MEGAWHOP PCR
PxylA
pHCMC04G
TTAAATCAAAGGGGGAAATGACAAATGGTCTAAACTAGTGATATCTAAAAATCA
L N Q R G K - Q M V - T S D I - K S

Figure S3

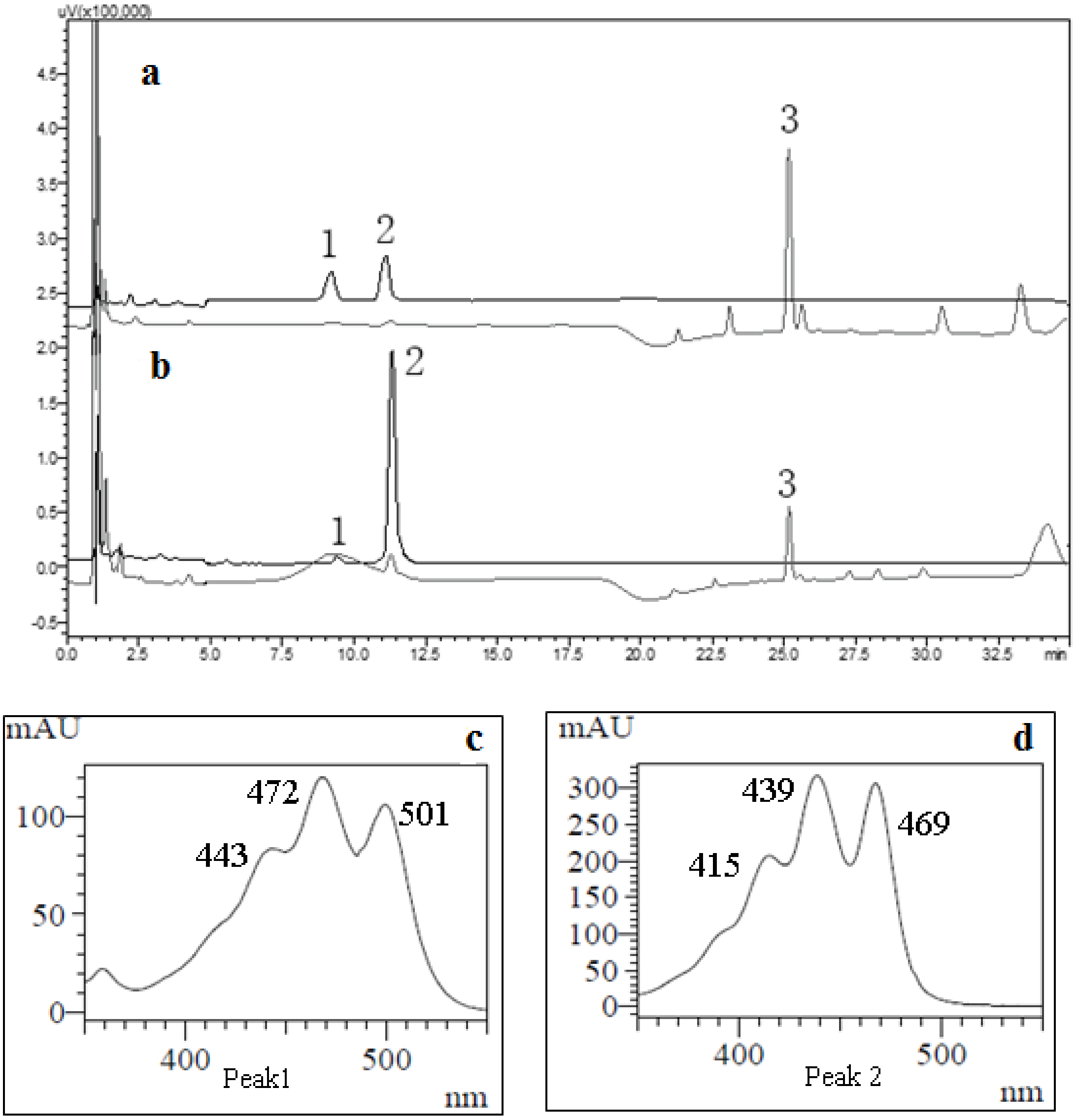

uV(x100,000)
a
b
c
d
1
2
3
mAU
472
501
443
Peak1
439
469
415
Peak 2
nm
min

Figure S4

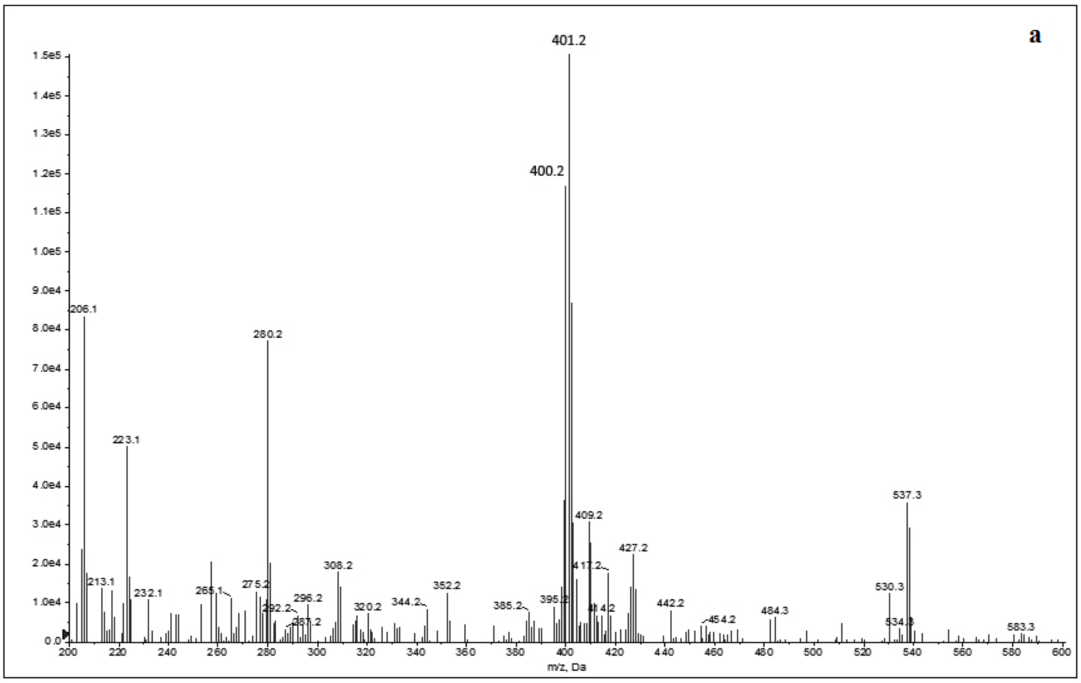
a
401.2
400.2
206.1
280.2
223.1
537.3
409.2
427.2
308.2
352.2
530.3
442.2
484.3
583.3
m/z, Da

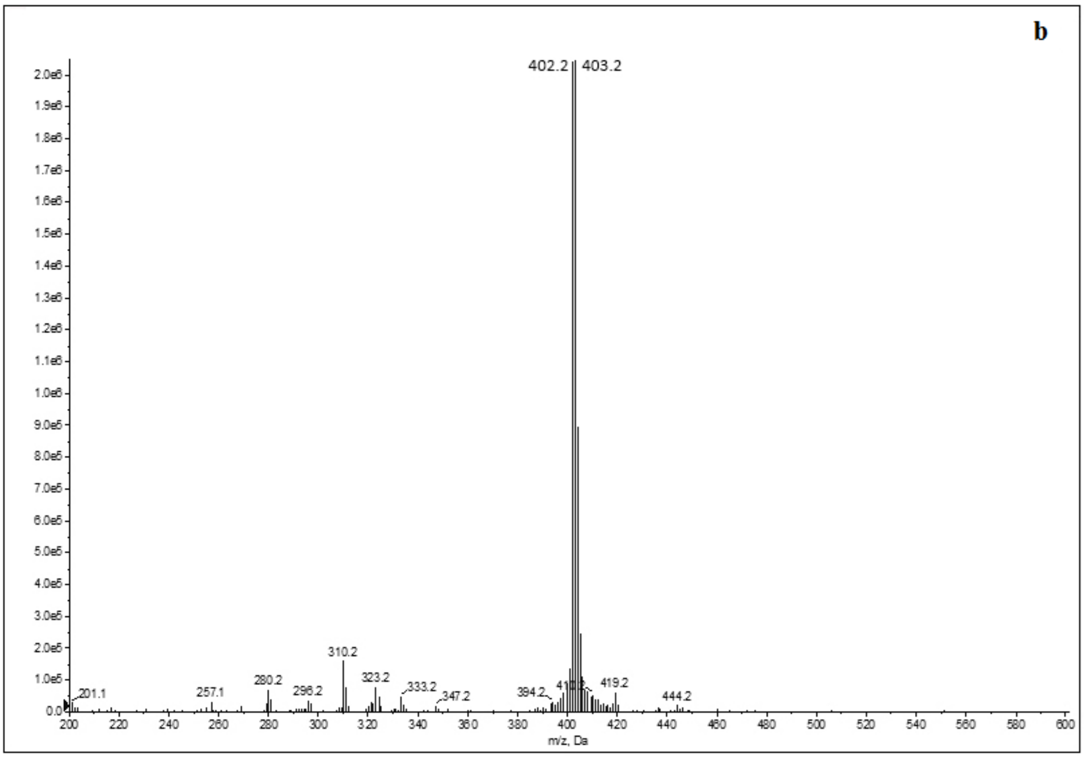
b
402.2
403.2
310.2
323.2
280.2
333.2
419.2
257.1
201.1
296.2
347.2
394.2
444.2
m/z, Da
